# A Follow-Up Study of the Supraaortic and Intracranial Vessels, Cerebrovascular Reactivity, Brain Vascular Lesions and Atrophy in Patients with Rheumatoid Arthritis

**DOI:** 10.3390/jcm15124691

**Published:** 2026-06-17

**Authors:** Attila Sas, Dávid Jónyer, Attila Valikovics, László Kostyál, Zsuzsanna Oláh, Katalin Hodosi, Zsófia Kardos, Csaba Oláh, Zoltán Szekanecz

**Affiliations:** 1 Doctoral School of Clinical Medicine, University of Debrecen, 4032 Debrecen, Hungary; 2 Department of Stroke-Neurology-Toxicology, Borsod-Abaúj-Zemplén County Central Hospital and University Teaching Hospital, 3526 Miskolc, Hungary; david.jonyer@gmail.com (D.J.); valikovics.idegtox@bazmkorhaz.hu (A.V.); 3 Faculty of Health Sciences, University of Miskolc, 3515 Miskolc, Hungary; dr.kardos.zsofia@gmail.com; 4 Department of Radiology, Borsod-Abaúj-Zemplén County Central Hospital and University Teaching Hospital, 3526 Miskolc, Hungary; kostyalfed@gmail.com; 5 Doctoral School of Health Sciences, University of Debrecen, 4032 Debrecen, Hungary; olahzsuzso@gmail.com; 6 Institute of Nursing Science and Diagnostic Imaging, University of Miskolc, 3515 Miskolc, Hungary; 7 Department of Internal Medicine, Faculty of Medicine, University of Debrecen, 4032 Debrecen, Hungary; hodosi@med.unideb.hu; 8 Department of Rheumatology, Borsod-Abaúj-Zemplén County Central Hospital and University Teaching Hospital, 3526 Miskolc, Hungary; 9 Department of Neurosurgery, Borsod-Abaúj-Zemplén County Central Hospital and University Teaching Hospital, 3526 Miskolc, Hungary; olahcs@gmail.com; 10 Mathias Institute, University of Tokaj, 3950 Sarospatak, Hungary; 11 Department of Rheumatology, Faculty of Medicine, University of Debrecen, 4032 Debrecen, Hungary; szekanecz.zoltan@med.unideb.hu

**Keywords:** rheumatoid arthritis, accelerated atherosclerosis, cerebrovascular reactivity, transcranial doppler, biologic treatment

## Abstract

**Background/Objectives:** Rheumatoid arthritis (RA) has been associated with accelerated atherosclerosis and cerebrovascular alterations. Our 2017 study compared 60 RA patients to healthy controls, assessing vascular, neurological, and cognitive parameters. The present study is a follow-up of these RA patients to evaluate disease progression and vascular changes over time, using their 2017 results as baseline. **Methods:** In 2023, we reassessed 43 of the original 60 RA patients using laboratory testing, carotid ultrasound, functional transcranial Doppler (TCD) and brain magnetic resonance imaging (MRI) examinations. Changes over time were analyzed within the same individuals. **Results:** Inflammatory markers and lipid profiles showed a trend toward improvement, though changes were not statistically significant, except for a significant increase in vitamin D (*p* < 0.001) and a decrease in Disease Activity Score in 28 Joints (DAS28) scores (*p* < 0.001). Carotid ultrasound revealed a significant increase in plaque burden (*p* = 0.022 on the right side and *p* = 0.008 on the left), while carotid intima media thickness (cIMT) showed a non-significant rise. TCD measurements indicated significantly increased pulsatility (*p* < 0.001 on the right, *p* = 0.001 on the left side) and resistance (*p* = 0.001 on the right, *p* = 0.012 on the left side) indices and reduced flow velocities (*p* < 0.001 on the right and *p* = 0.001 on the left side) in bilateral middle cerebral arteries (MCAs). The cerebrovascular reserve capacity was significantly lower on the right side overall (*p* = 0.013), with further decline noted in the methotrexate (MTX)-treated subgroup on the left side (*p* = 0.043). MRI findings showed non-significant numerical trends toward worsening lacunar small-vessel disease (*p* = 0.405) and cerebral atrophy (*p* = 0.063), with higher but stable lacunar infarction scores among MTX users (*p* = 0.023). **Conclusions:** Despite improved inflammatory control, RA patients demonstrated progressive vascular and hemodynamic alterations over time, while MRI changes should be interpreted as trends. These findings support multimodal vascular monitoring in RA.

## 1. Introduction

Rheumatoid arthritis (RA) is a chronic autoimmune disease that substantially increases cardiovascular (CV) risk through accelerated atherosclerosis [1]. While pro-inflammatory cytokines such as TNF-α, IL-1β, and IL-6 play a key role in endothelial dysfunction and plaque development [2], the main challenge is documenting how these mechanisms translate into measurable vascular changes in vivo.

Several imaging studies have demonstrated early vascular involvement in RA. Increased arterial ^18^F-FDG uptake was reported in RA compared to osteoarthritis, correlating with disease activity markers [3]. Moreover, tofacitinib reduced both synovial and vascular FDG uptake, highlighting PET/CT as a sensitive tool for monitoring treatment response [4]. Carotid ultrasound and intima media thickness (cIMT) have been widely studied as markers of subclinical atherosclerosis [5,6,7]. In our 2017 RA cohort, we found significantly increased IMT compared to healthy controls, confirming early vascular remodeling [8]. This aligns with other studies linking cIMT progression to systemic inflammation, particularly CRP and IL-6 [9]. Carotid plaque formation is also more frequent in RA, even in the absence of conventional risk factors, and plaques in this group are more prone to rupture [10,11,12]. These findings help explain the markedly increased risk of ischemic stroke and myocardial infarction, with RA patients experiencing 1.5–2 times higher stroke risk [13,14] and shortened life expectancy by 5–7 years due to CV complications [15,16,17,18].

Low-frequency ultrasound (<2 MHz) allows examination of the major basal cerebral arteries through the thin temporal bone [19]. Subsequent technical developments led to the widespread use of transcranial Doppler ultrasound (TCD), a non-invasive, portable, simple, and cost-effective method for real-time assessment of cerebral blood flow and functional testing [20]. Today, TCD and transcranial color Doppler (TCCD) are routinely applied for diagnostic, monitoring, and functional purposes, including assessment of cerebral ischemia risk, MCA flow dynamics, cerebrovascular reserve capacity, intracranial pressure, brain death, patent foramen ovale, and cerebral emboli [21,22].

Our group has also contributed to the relatively small body of literature on transcranial Doppler (TCD) in RA. In our comparative study, RA patients showed significantly higher resistance index (RI) and pulsatility index (PI), alongside reduced cerebrovascular reserve capacity (CRC), compared with healthy controls [8,23]. These results indicate stiffer intracranial arteries and impaired endothelial function, which may underlie the increased cerebrovascular risk in RA. Importantly, few prior studies have examined cerebral hemodynamics in RA, underscoring the novelty of our contribution. Brain MRI further expands vascular assessment. In our 2017 cohort of 41 RA patients who underwent MRI, almost half showed vascular lesions, more frequent among methotrexate-treated patients compared with those on biologics, though not statistically significant. Cerebral atrophy was observed in 24% [8]. MRI’s ability to detect both symptomatic and silent infarctions provides valuable prognostic information, as silent lesions predict future stroke and cognitive decline [24,25].

The aim of our present study was to compare the carotid ultrasound and TCD parameters, as well as the MRI findings, measured in our RA patients from the 2017 study with the results obtained in the current investigation [8]. Based on the vascular effects of RA detailed above, we expected to observe progressive atherosclerosis, along with its corresponding signs detectable by carotid ultrasound, TCD, and MRI. In addition, we compared RA patients across therapeutic groups in an exploratory manner to examine whether vascular and cerebrovascular trajectories differed according to dominant long-term antirheumatic treatment exposure, without inferring treatment superiority or inferiority from these observational subgroup analyses.

## 2. Materials and Methods

### 2.1. Study Design, Source Cohort and Ethical Approval

**Figure 1:**
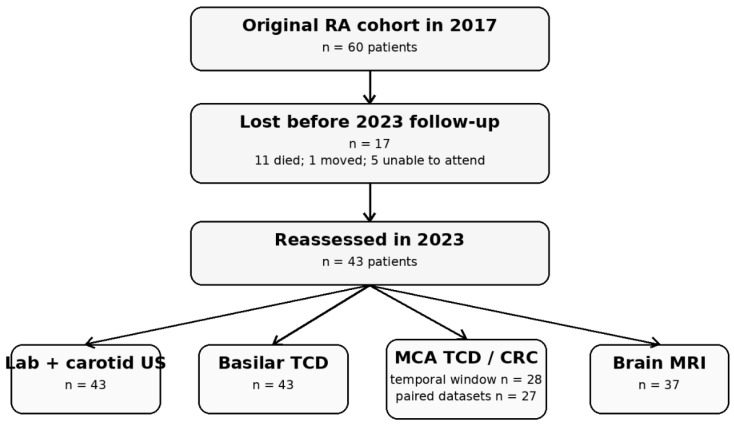
Patient flow diagram of the longitudinal RA cohort, including the original 2017 cohort, follow-up availability in 2023, loss to follow-up and investigation-specific sample sizes.

This investigation was designed as a longitudinal follow-up of our previously published 2017 RA cohort [8]. The baseline project enrolled 60 RA patients and healthy controls and included laboratory testing, carotid ultrasonography, TCD/TCCD assessment and brain MRI. In the present phase, we invited the original RA patients for repeated evaluation in 2023. Forty-three patients were available, provided written informed consent and completed the core follow-up protocol. Eleven patients had died during the interval, one patient had moved to another county, and five patients could not undergo follow-up examination because of substantial disability or technical/logistical reasons. The patient flow and investigation-specific sample sizes are summarized in Figure 1.

The 2017 measurements are referred to as Index 1 or baseline measurements, whereas the 2023 measurements are referred to as Index 2 or follow-up measurements. The main analysis compared the same patients’ 2023 results with their own 2017 data, thereby reducing between-subject variability. Patients were classified according to their dominant long-term antirheumatic treatment exposure during the follow-up period and their follow-up treatment status: MTX (*n* = 14), IFX (*n* = 14), or TCZ (*n* = 15). These groups were observational treatment categories and were not randomly assigned; therefore, subgroup analyses were considered exploratory and interpreted cautiously.

The study complied with the Declaration of Helsinki and was approved by the Regional Scientific and Research Ethics Committee (approval numbers 1046-63/2015 and BORS-04/2023; 17 January 2023). All participants provided written informed consent before the follow-up assessment.

### 2.2. Clinical and Laboratory Assessment

Demographic characteristics, disease duration, treatment history, smoking and alcohol consumption status, body mass index (BMI), rheumatoid factor (RF) and anti-citrullinated protein antibody (anti-CCP) status were recorded. Disease activity was assessed using the 28-Joint Disease Activity Score (DAS28). Laboratory follow-up included the erythrocyte sedimentation rate (ESR), C-reactive protein (CRP), lipid parameters (total cholesterol, HDL, LDL and triglycerides), beta-C-terminal telopeptide of type I collagen (beta-CTx), vitamin D and the relevant routine clinical parameters available from the clinical follow-up visit.

Laboratory and clinical results were analyzed for the whole cohort and, where informative, across treatment groups. A compact summary of the clinically most relevant longitudinal changes is presented in the main text, whereas the complete treatment group laboratory table is provided as Supplementary Table S2.

### 2.3. Carotid Ultrasound Protocol

All carotid examinations were performed by the same experienced neurosonographer (A.S.) using a GE VIVID S5 ultrasound system (GE Vingmed Ultrasound AS, Horten, Norway) with a 5 MHz linear-array transducer. Participants were examined in the supine position. Both carotid systems were evaluated using longitudinal and transverse B-mode imaging, color Doppler and spectral Doppler.

cIMT was measured on a 10 mm segment of the distal common carotid artery posterior wall, at least 5 mm below the carotid bifurcation, using dedicated software. Values were expressed in millimeters. All visible plaques were recorded and classified according to plaque composition and stenotic relevance. For longitudinal comparison, plaque score categories were collapsed into low plaque burden (0–1) versus higher plaque burden (2–5), where higher scores represented calcified, stenotic or soft plaques with greater presumed vascular relevance [26,27].

### 2.4. Transcranial Doppler and Functional Cerebrovascular Testing

TCD examinations were performed by the same neurosonographer (D.J.) using a DWL Multi-Dop T digital system with a 2 MHz probe and a DiaMon recorder (Compumedics Germany GmbH (DWL), Singen, Germany). The middle cerebral artery (MCA) was examined through the transtemporal window, typically at a depth of 50–55 mm. In patients with an insufficient conventional TCD signal, TCCD verification was performed using a GE VIVID S5 system with a 3ScRS probe to reduce the risk of false-negative insonation failure (GE Medical Systems, Chicago, IL, USA). Temporal acoustic window failure was defined when both TCD and TCCD failed to visualize flow signals at the expected arterial sites [23]. Patients without adequate temporal windows were excluded from MCA-specific TCD and CRC analyses but remained included in all other analyses for which data were available.

For each assessable MCA, resting mean flow velocity (RMV), resting pulsatility index (RPI) and resting resistance index (RRI) were recorded. Functional testing consisted of 30 s of voluntary hyperventilation followed by a 30 s breath-holding test. Hyperventilation-related and post-apnea/breath-hold mean velocities, pulsatility indices and resistance indices were documented. CRC was calculated from the breath-hold response as CRC = [(BFVpost-breath-hold − BFVrest)/BFVrest] × 100. CO_2_ levels were not continuously monitored during the examination. Basilar artery flow was assessed through the suboccipital transforaminal window. A temporal window was available in 28 of the 43 patients; complete paired MCA functional datasets suitable for longitudinal analysis were available in 27 patients.

### 2.5. Brain MRI Protocol and Lesion Scoring

Brain MRI examinations were performed on the same MAGNETOM Verio 3T scanner (Siemens, Munich, Germany) used in the baseline investigation. The examinations were conducted by the same radiologist (L.K.) and radiographer (Z.O.) and were evaluated by the same neurosurgeon/neuroradiologist (C.O.), increasing consistency between the baseline and follow-up assessments.

The analysis focused on vascular encephalopathy, lacunar lesions, non-lacunar ischemic lesions and cerebral atrophy. The MRI definitions were based on size-based criteria consistent with recognized standards for neuroimaging of cerebral small-vessel disease [24]. Lacunar lesions were defined as lesions below 15 mm and were scored on a 0–3 ordinal scale (0 = none, 1 = one lesion, 2 = few lesions, 3 = multiple lesions). Non-lacunar lesions larger than 15 mm were coded as absent or present. Cerebral atrophy was coded as 0 when absent or age-appropriate and 1 when advanced for age. For the present analysis, hemispheric distribution was not separately evaluated. The MRI reader was blinded to the time point, treatment group and clinical data during image evaluation.

### 2.6. Statistical Analysis

Statistical analysis was performed by the same medical statistician (K.H.) using IBM SPSS Statistics version 22 (IBM, Armonk, NY, USA). Continuous data are expressed as mean ± standard deviation (SD), and categorical variables are expressed as frequency and percentage. Paired longitudinal comparisons used paired two-tailed *t* tests or Wilcoxon signed-rank tests, depending on distribution and measurement level. Categorical variables were compared with chi-square or Fisher’s exact tests, as appropriate.

Associations between clinical, laboratory and vascular variables were explored using Spearman correlation analysis and stepwise multiple linear regression. TCD and carotid parameters were considered dependent variables, whereas age, disease duration, RF/anti-CCP positivity, MTX dose and duration, biologic therapy duration, DAS28, ESR, hsCRP, BMI and lipid parameters were considered candidate independent variables. Standardized beta coefficients and unstandardized B coefficients with 95% confidence intervals were calculated when applicable. Two-sided *p*-values below 0.05 were considered statistically significant. No formal correction for multiple testing was applied because the analyses were exploratory; therefore, *p*-values should be interpreted cautiously and in the context of consistency across related vascular, hemodynamic and imaging measures.

## 3. Results

### 3.1. Participant Flow and Systemic Disease Control

The follow-up cohort consisted of 43 female RA patients (Table 1). Their mean age increased from 59.44 ± 7.77 years at baseline to 67.79 ± 7.70 years at follow-up, and mean disease duration increased from 10.48 ± 6.26 to 18.72 ± 6.52 years. Treatment distribution at follow-up was MTX in 14 patients, IFX in 14 patients and TCZ in 15 patients. Smoking and alcohol consumption did not change significantly during the follow-up interval.

**Figure 2:**
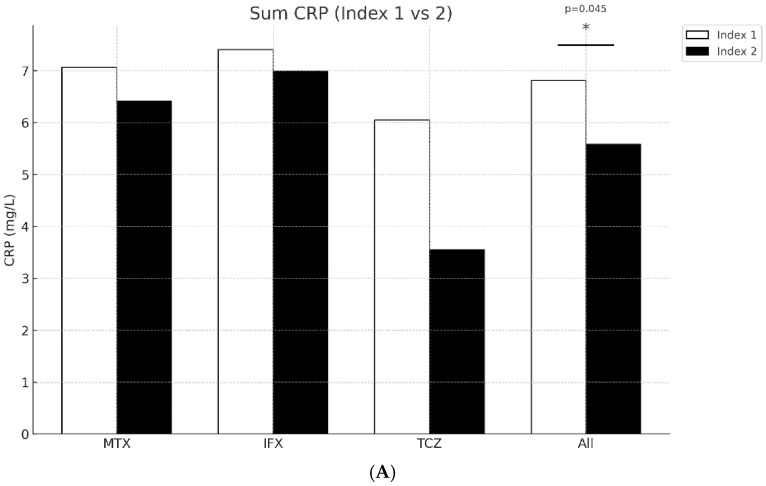

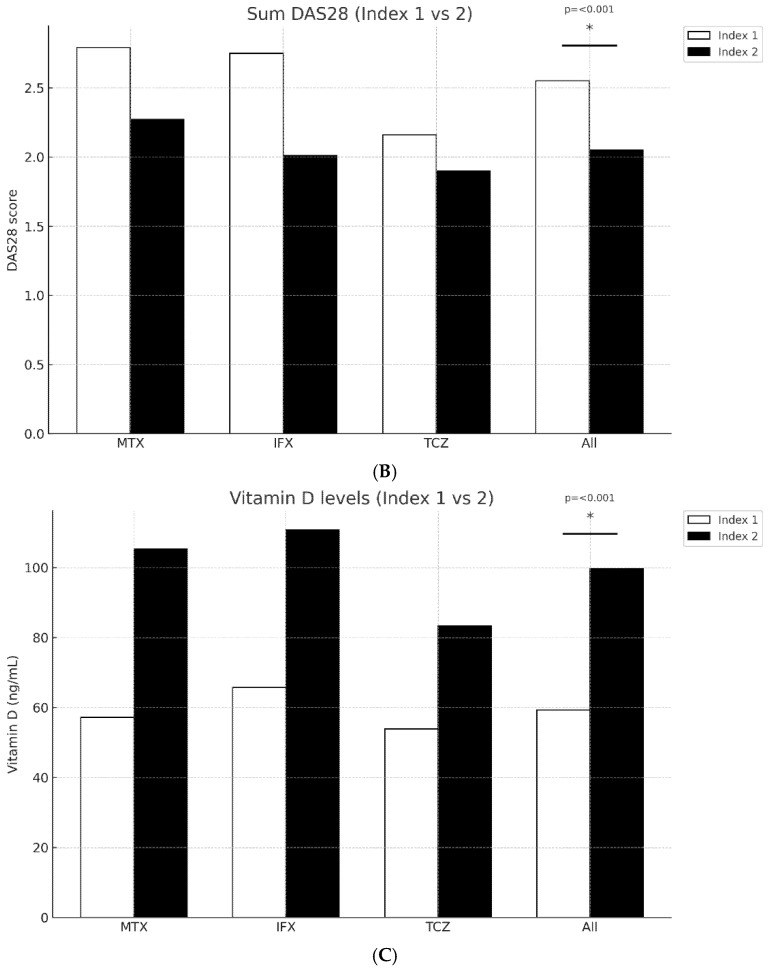
Key inflammatory and metabolic changes across treatment groups. (**A**) Sum of CRP levels, (**B**) DAS28 scores and (**C**) vitamin D levels at baseline (Index 1) and follow-up (Index 2). * means statistical significance.

Systemic disease activity improved despite the aging of the cohort. CRP decreased significantly (*p* = 0.045), DAS28 decreased significantly (*p* < 0.001), and vitamin D levels increased significantly (*p* < 0.001) (Figure 2). ESR, total cholesterol and LDL showed non-significant decreases, whereas HDL and triglyceride levels were higher at follow-up. MTX use decreased from 42 patients at baseline to 29 patients at follow-up, reflecting the broader use of biologic therapy during long-term care. The complete laboratory dataset is provided as Supplementary Table S2.

### 3.2. Carotid Ultrasound Findings

**Figure 3:**
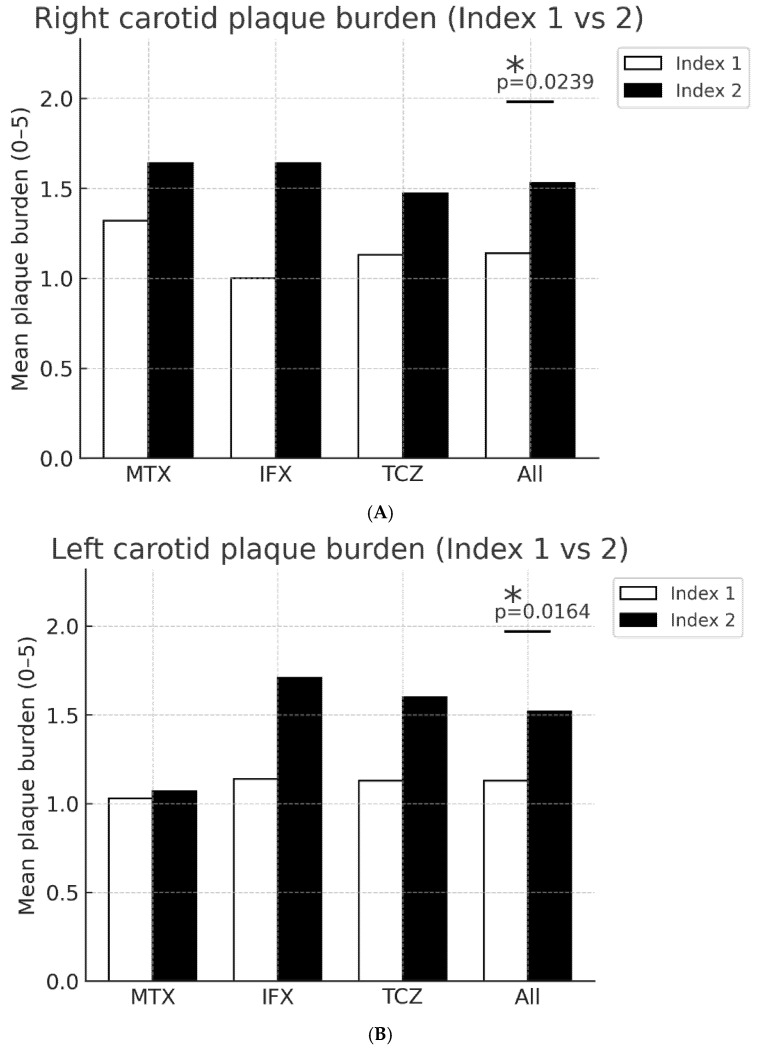

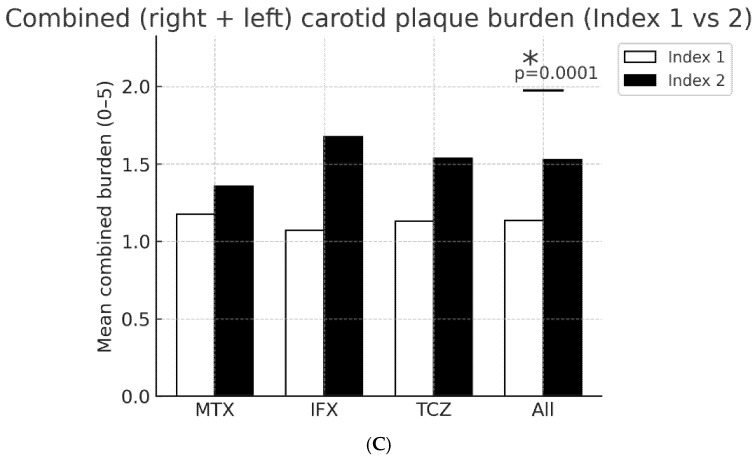
Carotid plaque burden progression. (**A**) Right carotid plaque burden, (**B**) left carotid plaque burden and (**C**) combined right plus left plaque burden, with significance marking for longitudinal changes. * means statistical significance.

All 43 patients underwent carotid ultrasound at follow-up. Compared with the 2017 baseline examination, plaque burden increased significantly in both the right and left carotid systems. The number of patients in the higher plaque score category (2–5) increased from 22 to 31 on the right side (*p* = 0.022) and from 19 to 30 on the left side (*p* = 0.008). The progression was driven mainly by an increase in calcified and calcified–stenotic plaques, whereas soft plaques were less frequent at follow-up (Table 2, Figure 3).

cIMT values increased slightly on both sides, but these changes were not statistically significant. Right cIMT increased from 0.758 ± 0.280 to 0.797 ± 0.166 mm (*p* = 0.131), and left cIMT increased from 0.788 ± 0.258 to 0.793 ± 0.146 mm (*p* = 0.739). No significant treatment group difference in carotid plaque or cIMT progression was detected. Detailed plaque subtype data are presented in Supplementary Table S3. Carotid plaque scores, cIMT and stenosis progression data are presented in Supplementary Table S4 and data across treatment groups in Supplementary Table S5.

### 3.3. Transcranial Doppler Findings and Cerebrovascular Reserve Capacity

Flow through the basilar artery could be measured through the transforaminal window in all 43 patients. A usable temporal window was present in 28 patients, and complete paired MCA functional datasets were available for 27 patients. In the whole paired TCD subgroup, both MCAs showed a consistent pattern of increased pulsatility and resistance indices with reduced mean flow velocities at follow-up.

On the right side, resting PI and RI were higher at follow-up (RPI *p* < 0.001; RRI *p* = 0.001), while resting mean velocity decreased significantly (*p* < 0.001). The same direction of change was observed after hyperventilation and after the apnea/breath-hold test. On the left side, resting PI and RI also increased (*p* = 0.001 and *p* = 0.012, respectively), and resting mean velocity decreased (*p* = 0.001) (Table 3). Hyperventilation and breath-hold values showed comparable deterioration. Detailed treatment-group TCD values are reported in Supplementary Table S6.

**Figure 4:**
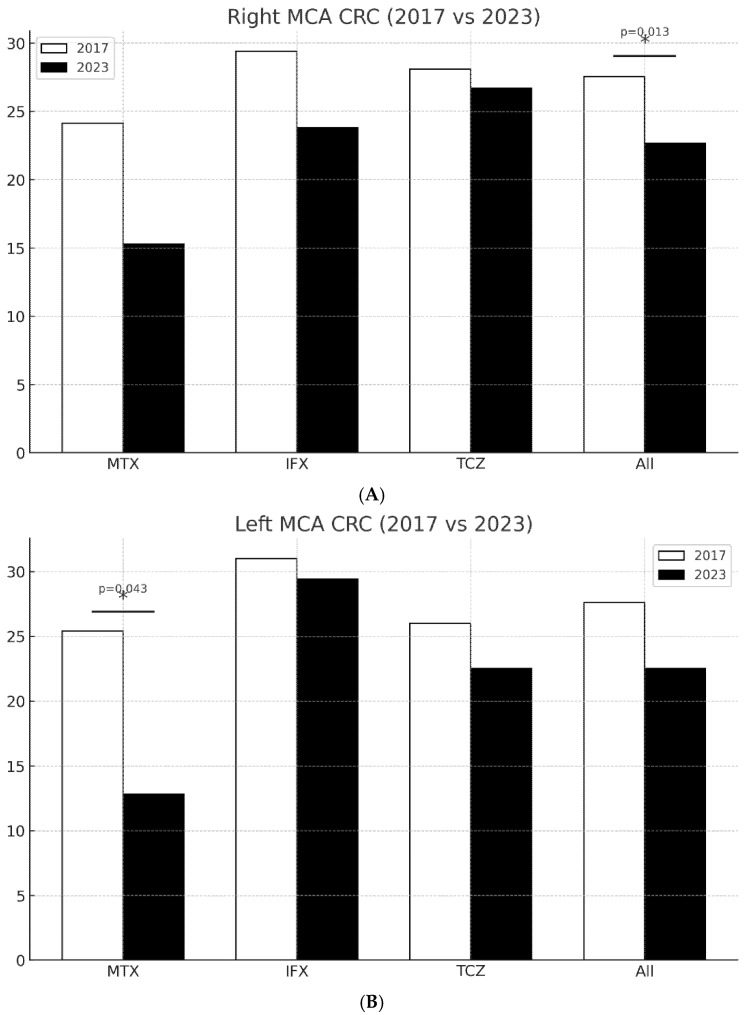
Cerebrovascular reserve capacity (CRC) in RA patients treated with MTX, IFX and TCZ and in all patients. (**A**) Right MCA CRC and (**B**) left MCA CRC at baseline and follow-up. * means statistical significance.

CRC analysis showed a significant right-sided decline in the complete paired TCD subgroup (27.56 ± 13.29% to 22.67 ± 16.74%, *p* = 0.013). Left-sided CRC declined from 27.60 ± 13.46% to 22.50 ± 19.33%, but the change did not reach statistical significance in the whole group (*p* = 0.144). In the MTX subgroup, both sides declined over time, with a significant left-sided decrease (25.40 ± 9.86% to 12.80 ± 6.94%, *p* = 0.043) and a near-significant right-sided decrease (*p* = 0.091) (Figure 4, Table 4). Basilar artery resting mean velocity was significantly lower at follow-up (*p* < 0.001), whereas basilar PI did not change significantly in the whole cohort.

### 3.4. Brain MRI Findings

Brain MRI was completed in 37 of the 43 follow-up patients. Six patients could not undergo MRI because of technical reasons, such as implants or claustrophobia. The same 37 patients had also participated in the MRI component of the baseline study, allowing paired longitudinal comparison.

Lacunar small-vessel disease showed a non-significant tendency toward worsening. The number of patients with no lacunar lesion decreased from 20 at baseline to 15 at follow-up. When lacunar scores were dichotomized as lower (0–1) versus higher (2–3), the higher-score category increased from 15 to 18 patients (*p* = 0.405). MTX-treated patients had higher lacunar scores than the IFX and TCZ groups at both baseline and follow-up, and this difference persisted over time (2017 *p* = 0.029; 2023 *p* = 0.023).

**Figure 5:**
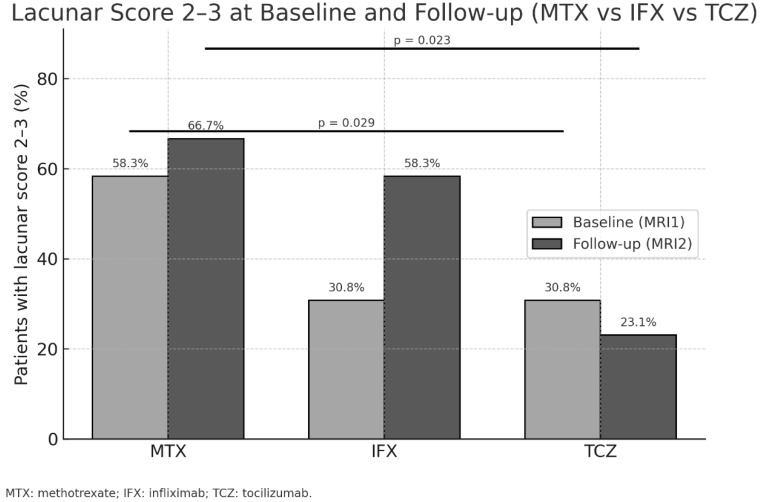
Combined baseline (MRI1) and follow-up (MRI2) lacunar score results. The figure shows the percentage of patients with high lacunar score (2–3) in each treatment group, with significant differences between treatment groups at baseline and follow-up.

Non-lacunar ischemic lesions were present in more patients at follow-up than at baseline (six versus four patients), but this change was not statistically significant. Cerebral atrophy showed a near-significant increase: the number of patients with atrophy increased from 10 to 13, while those without advanced atrophy decreased from 28 to 24 (*p* = 0.063). No significant treatment group difference was observed in the rate of atrophy progression (Table 5, Figure 5).

## 4. Discussion

### 4.1. Principal Findings

This longitudinal follow-up study provides a multimodal assessment of vascular and neuroimaging progression in RA patients who had previously undergone detailed carotid ultrasound, functional TCD/TCCD and brain MRI evaluation. The main finding is that improved systemic inflammatory control did not fully prevent vascular and cerebrovascular progression. During follow-up, CRP and DAS28 improved, whereas carotid plaque burden increased, MCA flow velocities decreased, MCA PI and RI increased, CRC declined and MRI showed trends toward worsening small-vessel disease and atrophy.

The combination of extracranial plaque progression, intracranial hemodynamic deterioration and structural MRI abnormalities supports the concept that vascular injury in patients with RA is systemic and persistent. These changes were not uniformly captured by cIMT alone, highlighting the importance of evaluating plaque burden, functional intracranial hemodynamics and brain imaging together.

### 4.2. Systemic Inflammation and Persistent Vascular Risk

The improvement in CRP and DAS28 suggests that contemporary RA management achieved better systemic disease control in this cohort. Nevertheless, atherosclerotic progression remained detectable. This apparent dissociation is clinically relevant. Vascular remodeling initiated by years of inflammation may persist even after inflammatory markers improve, and atherosclerosis can continue through endothelial dysfunction, oxidative stress, lipid exposure, vascular calcification and age-related arterial stiffening [2,5,17].

The significant increase in vitamin D probably reflects better supplementation and medical follow-up rather than a direct vascular effect in this observational study. Similarly, lipid values improved in some respects, but these favorable trends were not sufficient to eliminate plaque progression. These findings support the view that RA patients may require vascular surveillance even when joint disease activity appears well controlled.

### 4.3. Carotid Ultrasound Interpretation

Carotid plaque burden increased significantly on both sides, while cIMT did not change significantly. This pattern suggests that plaque assessment may be more sensitive than cIMT for detecting clinically relevant atherosclerotic progression in older RA patients. cIMT represents diffuse wall thickening, whereas plaque burden captures focal atherosclerotic disease and may better reflect accumulated vascular injury [15,18,26,27].

The progression was driven mainly by calcified and calcified–stenotic plaques. Calcification may indicate chronic plaque maturation, whereas the presence of stenotic plaques signals a potentially more advanced vascular phenotype. The absence of significant treatment group differences in plaque progression should be interpreted cautiously because the study was not powered for definitive treatment comparisons and treatment allocation was not randomized.

### 4.4. Intracranial Hemodynamics and Cerebrovascular Reserve

The TCD findings were consistent across both MCAs: the PI and RI increased, and mean flow velocities decreased at rest and after functional testing. A higher PI and RI can reflect increased downstream resistance, reduced arterial compliance or greater pulsatile transmission into the microcirculation. Lower flow velocities may indicate impaired perfusion reserve, luminal narrowing or altered autoregulatory adaptation [28,29,30]. In RA, these mechanisms are biologically plausible because chronic inflammation can impair nitric oxide-mediated vasodilation and promote arterial stiffness [31,32,33,34].

CRC declined significantly on the right side in the whole paired TCD subgroup and significantly on the left side in MTX-treated patients. Reduced CRC suggests a diminished capacity of cerebral resistance vessels to respond to CO2-mediated vasodilatory demand [35]. This may be clinically important because patients with impaired reserve may be more vulnerable to hypotension, embolic events or progressive small-vessel disease. The basilar artery finding of lower resting mean velocity further suggests that hemodynamic alterations were not limited to the anterior circulation.

### 4.5. MRI Findings and Neurovascular Relevance

MRI showed a tendency toward worsening lacunar small-vessel disease and a near-significant increase in cerebral atrophy. Although these changes did not all reach statistical significance, their direction was concordant with the TCD results. Increased resistance indices and reduced flow velocities may contribute to chronic hypoperfusion, and impaired cerebrovascular reserve may reduce the ability of the brain to compensate for vascular stress. Silent vascular lesions are clinically relevant because they are associated with future stroke risk and cognitive decline [24,25].

The persistently higher lacunar scores among MTX-treated patients may reflect baseline differences, disease severity, cumulative inflammatory burden or treatment selection bias rather than a direct harmful effect of MTX. Because this was an observational study, the treatment subgroup findings should be viewed as hypothesis-generating. Nonetheless, they support the need for larger longitudinal studies comparing cerebrovascular trajectories across antirheumatic treatment strategies [36,37,38].

### 4.6. Possible Clinical Applications and Future Research Directions

The present findings suggest several possible applications. First, carotid ultrasound could be used to identify RA patients with progressing extracranial atherosclerosis even when routine inflammatory markers improve. Second, TCD/TCCD with functional CRC testing may provide additional information on intracranial vascular resistance and reserve, especially in patients with vascular risk factors, neurological symptoms or MRI evidence of small-vessel disease [39,40]. Third, combining ultrasound and MRI may help to stratify patients who require more intensive cardiovascular prevention, neurological monitoring or individualized vascular risk management.

Future research should validate these observations in larger multicenter cohorts with standardized TCD protocols, automated or centralized MRI scoring, cognitive outcome measures and longer follow-up. Studies should also examine whether aggressive risk-factor control, biologic or targeted synthetic DMARD therapy, statin use, antiplatelet strategies in selected patients, and lifestyle interventions can modify TCD parameters, CRC, carotid plaque progression or MRI lesion burden. Integrating vascular imaging into RA follow-up may ultimately help define a clinically actionable neurovascular monitoring pathway. The therapeutic implications of vascular monitoring are particularly relevant in the current era of biologic and targeted synthetic DMARDs [41,42]. Cardiovascular safety concerns, especially those related to JAK inhibitors, have influenced prescribing behavior in RA and underscore the need for individualized risk assessment. Recent real-world evidence suggested that safety warnings affected JAK inhibitor prescribing patterns in RA, illustrating how vascular risk evaluation increasingly interacts with treatment selection [43].

### 4.7. Strengths and Limitations

The strengths of this study include the longitudinal within-patient design, the repeated use of the same imaging modalities, the involvement of the same experienced examiners, and the combined assessment of extracranial carotid atherosclerosis, intracranial functional hemodynamics, cerebrovascular reserve capacity, and brain MRI findings. This multimodal approach provides a detailed real-world picture of vascular and cerebrovascular changes in an elderly RA cohort during long-term follow-up.

The main limitation is the relatively small sample size, particularly for treatment subgroup and MCA TCD analyses. In addition, no age-matched longitudinal control group or population-based reference cohort was included. Therefore, although our findings demonstrate progression of vascular and cerebrovascular abnormalities within this RA cohort, the extent to which these changes exceed those expected from normal aging cannot be determined with certainty.

Another important limitation is attrition during follow-up. Of the original 60 RA patients, 43 were available for reassessment in 2023, while 11 patients had died, one patient had moved away, and five patients were unable to participate because of severe disability or technical/logistical reasons. The COVID-19 period may also have contributed to loss to follow-up in this elderly polymorbid cohort. Detailed causes of death, including specific cardiovascular or cerebrovascular causes, were not uniformly available. A detailed statistical comparison of baseline characteristics between reassessed participants and non-participants was limited by incomplete uniformly available data for the non-participant group. Therefore, survivorship bias cannot be excluded. Patients with more severe cardiovascular or neurological outcomes may have been less likely to participate in the follow-up examination, which may have led to an underestimation of the true long-term vascular and cerebrovascular progression in the original cohort.

The study included only women at follow-up, limiting the generalizability of the findings to men with RA. Treatment groups were observational and not randomized, therapies may have changed over time, and confounding by indication is possible. Therefore, comparisons between MTX, IFX, and TCZ subgroups should be interpreted as exploratory and hypothesis-generating rather than as evidence for the superiority or inferiority of any treatment. Unmeasured confounders, such as cumulative steroid exposure, vascular medication use, comorbidities, and detailed lifetime disease activity, may also have influenced vascular outcomes.

No formal correction for multiple testing was applied, which should be considered when interpreting isolated significant findings. Temporal acoustic window limitations reduced the number of complete paired MCA TCD datasets. In addition, CO_2_ levels were not continuously monitored during the CRC protocol; therefore, breath-holding and hyperventilation responses may have been influenced by patient cooperation and physiological variability. Although MRI evaluation used predefined criteria and the reader was blinded to time point, treatment group, and clinical data, future multicenter studies should consider centralized or automated MRI scoring to further improve standardization.

## 5. Conclusions

In this longitudinal cohort of patients with rheumatoid arthritis, systemic inflammatory control improved during follow-up, as reflected by lower disease activity and more favorable inflammatory parameters. However, despite this apparent improvement, several vascular markers showed progression over time, while neuroimaging markers showed non-significant trends toward deterioration. Carotid plaque burden increased significantly, while TCD/TCCD measurements demonstrated higher intracranial vascular resistance, lower flow velocities in the MCAs, and a decline in cerebrovascular reserve capacity. Brain MRI findings suggested non-significant tendencies toward worsening lacunar small-vessel disease and cerebral atrophy. Together, these results indicate that neurovascular risk in patients with RA may persist or progress even when conventional markers of systemic inflammation improve.

The findings support the concept that cerebrovascular involvement in RA is multifactorial and may not be fully captured by routine clinical or laboratory follow-up alone. Persistent endothelial dysfunction, vascular remodeling, arterial stiffness, and cumulative inflammatory burden may all contribute to long-term vascular progression. Therefore, RA patients—particularly those with long-standing disease or additional cardiovascular risk factors—may benefit from a more comprehensive vascular monitoring strategy.

A multimodal approach combining carotid ultrasound, functional TCD/TCCD assessment, and brain MRI may help detect subclinical vascular progression before clinically evident cerebrovascular events occur. Carotid ultrasound provides information on extracranial atherosclerotic burden, TCD/TCCD allows functional assessment of intracranial hemodynamics and cerebrovascular reserve, and MRI can identify silent ischemic lesions and structural brain changes. The combined use of these methods may improve risk stratification and may help identify patients who require closer neurological and cardiovascular follow-up.

Larger, multicenter, prospective studies are needed to confirm these findings and to clarify how vascular imaging and functional cerebrovascular testing should be incorporated into routine RA care. Future research should also determine whether intensified cardiovascular prevention, optimized anti-inflammatory treatment, or targeted neurovascular monitoring can slow cerebrovascular progression and improve long-term neurological outcomes in patients with RA.

## Figures and Tables

**Table 1:** Cohort flow and key systemic longitudinal findings in the RA follow-up cohort.

Variable	Baseline/Index 1 (2017)	Follow-Up/Index 2 (2023)	*p*-Value/Comment
Patients included	60 original RA patients	43 re-examined RA patients	11 died; 1 moved; 5 unable to attend
Treatment groups at follow-up	MTX *n* = 20; IFX *n* = 20; TCZ *n* = 20	MTX *n* = 14; IFX *n* = 14; TCZ *n* = 15	Observational treatment groups
Age (years)	59.44 ± 7.77	67.79 ± 7.70	Expected follow-up increase
Disease duration (years)	10.48 ± 6.26	18.72 ± 6.52	Expected follow-up increase
BMI (kg/m^2^)	27.69 ± 5.11	27.58 ± 5.52	No relevant change
CRP (mg/L)	5.61 ± 8.70	4.33 ± 7.39	*p* = 0.400
Sum CRP	6.81 ± 6.09	5.58 ± 8.04	*p* = 0.045
DAS28	2.42 ± 0.70	Lower at follow-up	*p* < 0.001
Vitamin D (nmol/L)	59.23 ± 27.24	99.60 ± 46.82	*p* < 0.001
RF/anti-CCP positivity	Recorded at baseline	Unchanged	Stable serological status

Values are mean ± SD unless otherwise stated. Detailed treatment-group demographic and laboratory results are provided in Supplementary Tables S1 and S2.

**Table 2:** Main carotid ultrasound progression results.

Parameter	Baseline/Index 1 (2017)	Follow-Up/Index 2 (2023)	*p*-Value
Right plaque score 0–1/2–5	21/22	12/31	0.022
Left plaque score 0–1/2–5	24/19	12/30	0.008
Right cIMT (mm)	0.758 ± 0.280	0.797 ± 0.166	0.131
Left cIMT (mm)	0.788 ± 0.258	0.793 ± 0.146	0.739
Right carotid stenosis (%)	2.33 ± 10.88	5.35 ± 17.23	0.102
Left carotid stenosis (%)	1.40 ± 9.15	2.33 ± 10.65	1.000

cIMT: carotid intima media thickness. Plaque score categories were dichotomized as 0–1 versus 2–5 for longitudinal comparison.

**Table 3:** Key paired TCD parameter changes in all patients with complete MCA datasets.

MCA Parameter	Baseline/Index 1	Follow-Up/Index 2	*p*-Value
Right RPI	0.908 ± 0.187	1.062 ± 0.229	<0.001
Right RRI	0.582 ± 0.069	0.639 ± 0.086	0.001
Right RMV	65.74 ± 10.96	48.56 ± 11.79	<0.001
Right AAPI	0.816 ± 0.156	1.028 ± 0.228	<0.001
Right AARI	0.552 ± 0.077	0.645 ± 0.086	<0.001
Right AAMV	82.52 ± 14.68	54.78 ± 20.65	<0.001
Left RPI	0.896 ± 0.191	1.003 ± 0.200	0.001
Left RRI	0.580 ± 0.076	0.620 ± 0.079	0.012
Left RMV	65.53 ± 8.73	48.84 ± 10.38	0.001
Left AAPI	0.860 ± 0.185	1.001 ± 0.244	0.007
Left AARI	0.578 ± 0.070	0.617 ± 0.088	0.022
Left AAMV	81.23 ± 12.48	53.62 ± 18.63	<0.001

RPI/RRI/RMV: resting pulsatility/resistance index and mean velocity; AAPI/AARI/AAMV: after-apnea pulsatility/resistance index and mean velocity. Full resting, hyperventilation and after-apnea values are available in Supplementary Table S7.

**Table 4:** Cerebrovascular reserve capacity (CRC) in the treatment groups and in all patients with complete paired MCA data.

CRC Parameter	MTX	IFX	TCZ	All Patients	Longitudinal *p*-Value of All Patients
Right MCA CRC 2017	24.14 ± 11.28	29.40 ± 17.44	28.10 ± 10.41	27.56 ± 13.29	-
Right MCA CRC 2023	15.29 ± 14.95	23.80 ± 22.13	26.70 ± 10.46	22.67 ± 16.74	0.013
Left MCA CRC 2017	25.40 ± 9.86	31.00 ± 20.34	26.00 ± 8.00	27.60 ± 13.46	-
Left MCA CRC 2023	12.80 ± 6.94	29.43 ± 25.38	22.50 ± 17.86	22.50 ± 19.33	0.144
Within-group MTX *p*-value	Right: 0.091; left: 0.043	-	-	-	-

Values are mean ± SD. CRC: cerebrovascular reserve capacity; MCA: middle cerebral artery; MTX: methotrexate; IFX: infliximab; TCZ: tocilizumab.

**Table 5:** Summary of longitudinal brain MRI findings.

MRI Variable	Baseline/Index 1	Follow-Up/Index 2	*p*-Value/Comment
Patients with completed MRI	38/43	37/43	Paired MRI data in 37 patients
Lacunar lesion score 0/1/2/3	20/3/8/7	15/4/7/11	Tendency toward worsening
Lacunar score 0–1/2–3	23/15	19/18	0.405
High lacunar score in MTX vs. IFX vs. TCZ	*p* = 0.029	*p* = 0.023	Persistently higher in MTX group
Non-lacunar lesion present/absent	4/34	6/31	0.500
Atrophy present/absent	10/28	13/24	0.063

Lacunar lesions were scored from 0 to 3. Non-lacunar lesions and atrophy were coded as present/absent. Detailed treatment group MRI findings are provided in Supplementary Table S9.

## Data Availability

The data presented in this study are available on reasonable request from the corresponding author.

## Supplementary Materials

The following supporting information can be downloaded at https://www.mdpi.com/article/10.3390/jcm15124691/s1, Table S1: Detailed demographic characteristics of RA patients across treatment groups; Table S2: Complete laboratory parameters in RA patients across treatment groups; Table S3: Carotid plaque characteristics across treatment groups; Table S4: Carotid plaque scores, intima media thickness and stenosis progression across treatment groups; Table S5: Intima media thickness and stenosis progression across treatment groups; Table S6: Complete transcranial Doppler flow parameters across treatment groups; Table S7: TCD parameter changes with *p*-values in all patients; Table S8: Complete cerebrovascular reserve capacity values in RA patients; Table S9: Detailed brain MRI findings at baseline and follow-up.

## Abbreviations

AAMV	after apnea mean velocity
AAPI	after apnea pulsatility index
AARI	after apnea resistance index
AHMV	after hyperventilation mean velocity
AHPI	after hyperventilation pulsatility index
AHRI	after hyperventilation resistance index
anti-CCP	anti-filagrin
APCAs	anti-citrullinated protein antibodies
Beta CTx	beta-C-terminal telopeptide of type I collagen
BMI	body mass index
CCA	common carotid artery
CBF	cerebral blood flow
cIMT	carotid Intima Media Thickness
CO_2_	carbon dioxide
CPP	cerebral perfusion pressure
CRC	cerebrovascular reserve capacity
CRP	C-reactive protein
CT	computer tomography
CV	cardiovascular
DAS28	Disease Activity Score in 28 Joints
DMARDs	disease-modifying antirheumatic drugs
ECA	external carotid artery
ESR	erythrocyte sedimentation rate
F-FDG	F-18 fluorodeoxyglucose
F-FDG PET	F-18 fluorodeoxyglucose positron emission tomography
HDL	high-density lipoprotein
ICA	internal carotid artery
IFX	infliximab
JAK	Janus kinase
IL-1β	interleukin-1 beta
IL-6	interleukin-6
IMT	intima media thickness
LDL	low-density lipoprotein
MCA	middle cerebral artery
Mhz	megahertz
MMPs	matrix metalloproteinases
MRI	magnetic resonance imaging
MTX	methotrexate
OA	osteoarthritis
OPG	osteoprotegerin
pCO_2_	partial CO_2_ pressure
RA	rheumatoid arthritis
RF	rheumatic factor
RMV	resting mean velocity
RPI	resting pulsatility index
RRI	resting resistance index
TAW	temporal acoustic window
TAWF	temporal acoustic window failure
TC	total cholesterol
TCD	transcranial doppler
TCCD	transcranial color doppler
TCZ	tocilizumab
TG	trigliceride
Th 1	T-helper cell 1
Th17	T-helper cell 17
TNF-α	tumor necrosis factor-alpha
US	ultrasound
VCAM-1	vascular cell adhesion molecule-1
