# Supplementary material for: A Follow-Up Study of the Supraaortic and Intracranial Vessels, Cerebrovascular Reactivity, Brain Vascular Lesions and Atrophy in Patients with Rheumatoid Arthritis
Source: J Clin Med. 2026 Jun 17;15(12):4691. doi: 10.3390/jcm15124691 (PMC13302623; doi:10.3390/jcm15124691)
Supplement: Supplementary file 1 [file jcm-15-04691-s001.zip › jcm-4361783-supplementary.pdf]

Supplementary Tables

A follow-up study of the supraaortic and intracranial vessels, cerebrovascular reactivity, brain vascular lesions and atrophy in patients with rheumatoid arthritis

Table S1. Detailed demographic characteristics of RA patients across treatment groups.

	MTX / (n=14)	IFX / (n=14)	TCZ / (n=15)	All patients / (n=43)	p MTX vs. IFX vs. TCZ	p MTX vs. IFX	p MTX vs. TCZ	p IFX vs TCZ
Age1	60.21±8.69	59.57±7.53	58.60±7.55	59.44±7.77	0.813	0.769	0.621	0.591
Age2	68.79±8.55	68.14±7.49	66.53±7.41	67.79±7.70	0.636	0.874	0.477	0.400
Education in years	13.07±1.94	10.86±3.16	10.73±2.89	11.53±2.86	0.014	0.031	0.007	0.880
BMI1	25.41±5.24	28.74±5.05	28.84±4.64	27.69±5.11	0.065	0.056	0.037	0.914
BMI2	25.42±5.26	28.89±6.39	28.38±4.54	27.58±5.52	0.146	0.125	0.070	0.949
Disease duration1	10.61±7.78	9.21±3.96	11.53±6.62	10.48±6.26	0.648	0.701	0.683	0.310
Disease duration2	19.36±8.40	17.21±3.96	19.53±6.62	18.72±6.52	0.648	0.701	0.780	0.310
Baseline therapy duration in years1	6.89±4.68	6.79±2.78	7.14±4.37	6.94±3.93	0.962	0.804	0.910	0.910
Baseline therapy duration in years2	14.77±9.02	15.50±2.02	13.67±5.51	14.96±6.36	0.576	0.376	0.900	0.448
MTX dosage1	13.75±3.77	13.93±4.78	19.64±2.37	15.77±4.60	<0.001	0.982	<0.001	0.002
MTX dosage2	15.58±4.35	14.38±5.24	18.33±2.89	15.36±4.65	0.456	0.611	0.364	0.295
Biologic treatment duration in years1	-	5.43±1.87	2.93±1.53	4.14±2.100	0.002	-	-	0.001
Biologic treatment duration in years2	-	12.71±2.09	11.87±2.00	11.59±2.95	0.012	-	-	0.354
smoker1 / non-smoker1	3 / 11	1 / 13	4 / 11	8 / 35	0.381	0.596	1.000	0.329
smoker2 / non smoker2	3 / 11	1 / 13	3 / 12	7 / 36	0.527	1.000	1.000	0.598
p				1.000				
alcohol consumption1 / non -alcohol consumption1	2 / 12	3 / 11	0 / 15	5 / 38	0.185	1.000	0.224	0.100
alcohol consumption2 / non-alcohol consumption2	1 / 13	2 / 12	0 / 15	3 / 40	0.320	1.000	0.483	0.224
p				0.500				

Abbreviations: index 1: baseline study; index 2: follow-up study; MTX: methotrexate; IFX:infliximab; TCZ: tocilizumab; BMI: body mass index

Table S2. Complete laboratory parameters in RA patients across treatment groups.

	MTX / (n=14)	IFX / (n=14)	TCZ / (n=15)	All patients / (n=43)	p MTX vs. IFX vs. TCZ	p MTX vs. IFX	p MTX vs. TCZ	p IFX vs TCZ
ESR1	17.86±13.40	23.64±15.31	9.13±8.36	16.70±13.72	0.002	0.285	0.008	0.001
ESR2	15.57±11.02	16.29±10.13	12.73±20.67	14.81±14.64	0.030	0.982	0.023	0.023
p				0.408				
Sum ESR1	19.56±13.85	21.89±10.91	12.87±11.82	17.95±12.53	0.021	0.402	0.052	0.008
Sum ESR2	17.86±12.89	21.38±13.96	11.12±12.67	16.63±13.58	0.018	0.720	0.046	0.005
p				0.472				
CRP1	8.91±13.12	4.76±4.97	3.32±5.18	5.61±8.70	0.047	0.352	0.029	0.063
CRP2	6.16±4.99	5.29±11.54	1.72±2.36	4.33±7.39	0.034	0.077	0.012	0,425
p				0.400				
Sum CRP1	7.06±4.11	7.40±8.21	6.05±5.53	6.81±6.09	0.572	0.402	0.363	0.880
Sum CRP2	6.41±4.21	6.99±12.53	3.55±4.61	5.58±8.04	0.108	0.116	0.046	0.747
p				0.045				
DAS28_1	2.61±0.79	2.53±0.62	2.14±0.64	2.42±0.70	0.264	0.874	0.217	0.134
DAS28_2	2.19±0.47	1.95±0.92	1.99±1.19	2.04±0.90	0.056	0.077	0.020	0.813
p				0.003				
Sum DAS28_1	2.79±0.79	2.75±0.50	2.16±0.72	2.55±0.72	0.045	0.943	0.058	0.020
Sum DAS28_2	2.27±0.43	2.01±0.73	1.90±0.75	2.05±0.66	0.064	0.061	0.033	0.652
p				<0.001				
TC1	5.22±0.70	5.54±0.95	5.83±1.34	5.541.05	0.289	0.430	0.118	0.451
TC2	5.47±1.09	5.23±0.88	5.62±1.24	5.45±1.07	0.774	0.720	0.964	0.425
p				0.861				
HDL1	1.46±0.40	1.58±0.37	1.59±0.48	1.55±0.42	0.772	0.462	0.648	0.949
HDL2	1.68±0.25	1.53±0.34	1.76±0.34	1.66±0.32	0.194	0.193	0.719	0.093
p				0.060				
LDL1	2.84±0.72	3.34±0.83	3.60±1.32	3.29±1.04	0.166	0.085	0.126	0.683
LDL2	3.12±1.05	3.05±0.85	3.30±1.05	3.16±0.97	0.831	0.820	0.683	0.591
p				0.909				
TG1	1.27±0.66	1.27±0.66	1.55±0.60	1.37±0.64	0.232	0.720	0.185	0.134
TG2	1.32±0.42	1.65±0.71	1.32±0.48	1.43±0.56	0.308	0.169	0.928	0.217
p				0.406				
Oszteokalcin1	18.09±7.55	19.26±6.82	18.44±5.99	18.66±6.58	0.751	0.508	0.648	0.720
Oszteokalcin2	18.15±9.93	20.66±10.00	18.14±6.14	19.20±8.65	0.791	0.585	0.693	0.685
p				0.835				
beta CTx1	0.34±0.11	0.32±0.13	0.29±0.17	0.31±0.14	0.502	0.752	0.343	0.350
beta CTx2	0.33±0.14	0.40±0.29	0.32±0.22	0.35±0.23	0.758	0.931	0.648	0.488
p				0.110				
D vitamin1	57.16±21.05	65.70±2.00	53.85±26.48	59.23±27.24	0.613	0.585	0.648	0.375
D vitamin2	105.33±38.12	110.68±53.96	83.25±43.26	99.60±46.82	0.182	0.931	0.101	0.141
p				<0.001				
tCalcium1	2.35±0.12	2.35±0.12	2.42±0.25	2.37±0.18	0.935	0.886	0.976	0.685
tCalcium2	2.34±0.13	2.35±0.35	2.36±0.08	2.35±0.22	0.330	0.312	0.648	0.169
p				0.733				
phosphate1	1.12±0.13	1.04±0.11	1.09±0.19	1.08±0.15	0.502	0.259	0.879	0.430
phosphate2	1.11±0.12	1.16±0.40	1.09±0.16	1.13±0.27	0.962	0.752	0.927	0,943
p				0.057				

Abbreviations: Index 1: previous study; Index 2: present study; MTX: methotrexate; IFX: infliximab; TCZ: tocilizumab; ESR: erythrocyte sedimentation rate; CRP: C-reactive protein; DAS28: disease activity score in 28 joints; TC: total cholesterol; HDL: high density lipoprotein; LDL: low density lipoprotein; TG: triglyceride; beta CTx: beta-C-terminal telopeptide of type I collagen.

Table S3. Carotid plaque characteristics across treatment groups.

	MTX / (n=14)	IFX / (n=14)	TCZ / (n=15)	All patients / (n=43)
right carotid plaque1 / 0 no / 1 fibrotic / 2 calcified / 3 calcified-stenotic / 4 soft / 5 soft-stenotic	6 / 0 / 7 / 0 / 1 / 0	7 / 0 / 7 / 0 / 0 / 0	8 / 0 / 4 / 1 / 1 / 1	21 / 0 / 18 / 1 / 2 / 1
left carotid plaque1 / 0 no / 1 fibrotic / 2 calcified / 3 calcified-stenotic / 4 soft / 5 soft-stenotic	8 / 0 / 5 / 0 / 0 / 1	6 / 0 / 8 / 0 / 0 / 0	7 / 3 / 4 / 1 / 0 / 0	21 / 3 / 17 / 1 / 0 / 1
right carotid plaque2 / 0 no / 1 fibrotic / 2 calcified / 3 calcified-stenotic / 4 soft / 5 soft-stenotic	5 / 0 / 8 / 1 / 0 / 0	3 / 0 / 10 / 1 / 0 / 0	3 / 1 / 9 / 2 / 0 / 0	11 / 1 / 27 / 4 / 0 / 0
left carotid plaque2 / 0 no / 1 fibrotic / 2 calcfied / 3 calcified-stenotic / 4 soft / 5 soft-stenotic	5 / 0 / 8 / 1 / 0 / 0	2 / 0 / 12 / 0 / 0 / 0	5 / 0 / 9 / 1 / 0 / 0	12 / 0 / 29 / 2 / 0 / 0

Abbreviations: MTX: methotrexate; IFX: infliximab; TCZ: tocilizumab

Table S4. Carotid plaque scores, intima-media thickness and stenosis progression across treatment groups.

	MTX / (n=14)	IFX / (n=14)	TCZ / (n=15)	All patients / (n=43)	p MTX vs. IFX vs. TCZ	p MTX vs. IFX	p MTX vs. TCZ	p IFX vs TCZ
right carotid plaque score1 / 0-1 / 2-5	6 / 8	7 / 7	8 / 7	21 / 22	0.316	1.000	0.715	0.715
right carotid plaque score2 / 0-1 / 2-5	5 / 9	3 / 11	4 / 11	12 / 31	0.695	0.678	0.700	1.000
p				0.022				
left carotid plaque score1 / 0-1 / 2-5	8 / 6	6 / 8	10 / 5	24 / 19	0.291	0.706	0.710	0.272
left carotid plaque score2 / 0-1 / 2-5	5 / 9	2 / 12	5 / 10	12 / 30	0.380	0.384	1.000	0.390
p				0.008				

Abbreviations: index 1: baseline study; index 2: follow-up study; MTX: methotrexate; IFX:infliximab; TCZ: tocilizumab

Table S5. Intima-media thickness and stenosis progression across treatment groups.

	MTX (n=14)	IFX (n=14)	TCZ (n=15)	All patients (n=43)	p MTX vs. IFX vs. TCZ	p MTX vs. IFX	p MTX vs. TCZ	p IFX vs. TCZ
Right carotid IMT1	0.750±0.268	0.764±0.227	0.760±0.346	0.758±0.280	0.804	0.982	0.683	0.505
Right carotid IMT2	0.765±0.192	0.847±0.183	0.781±0.115	0.797±0.166	0.906	0.701	0.880	0.747
p				0.131				
Right carotid stenosis1	0	0	6.67±18.00	2.33±10.88	0.148	1.000	0.561	0.561
Right carotid stenosis2	4.29±16.04	4.29±16.04	7.33±20.17	5.35±17.23	0.806	1.000	0.780	0.780
p				0.102				
Left carotid IMT1	0.750±0.263	0.793±0.281	0.820±0.246	0.788±0.258	0.760	0.769	0.477	0.683
Left carotid IMT2	0.769±0.157	0.807±0.169	0.802±0.118	0.793±0.146	0.642	0.482	0.400	0.813
p				0.739				
Left carotid stenosis1	4.29±16.04	0	0.020±0.077	1.40±9.15	0.607	0.769	0.983	0.780
Left carotid stenosis2	3.57±13.36	0	3.33±12.91	2.33±10.65	0.609	0.769	0.983	0.780
p				1.000				

Abbreviations: index 1: baseline study; index 2: follow-up study; MTX: methotrexate; IFX:infliximab; TCZ: tocilizumab; IMT: intima-media thickness

Table S6. Complete transcranial Doppler flow parameters across treatment groups.

	MTX / (n=7)	IFX / (n=10)	TCZ / (n=10)	All patients / (n=27)	p MTX vs. IFX vs. TCZ	p MTX vs. IFX	p MTX vs. TCZ	p IFX vs TCZ
Right RPI1	0.920±0.113	0.923±0.223	0.886±0.205	0.908±0.187	0.880	0.740	0.669	0.853
Right RPI2	1.033±0.240	1.059±0.208	1.085±0.261	1.062±0.229	0.891	0.740	0.669	0.971
p				<0.001				
Right RRI1	0.584±0.039	0.590±0.076	0.573±0.083	0.582±0.069	0.845	0.887	0.601	0.684
Right RRI2	0.639±0.097	0.652±0.088	0.627±0.086	0.639±0.086	0.846	0.813	0.813	0.529
p				0.001				
Right RMV1	69.29±8.60	63.50±12.92	65.50±10.73	65.74±10.96	0.425	0.230	0.669	0.436
Right RMV2	55.57±12.37	46.10±13.89	46.10±7.42	48.56±11.79	0.425	0.193	0.109	0.912
p				<0.001				
Right AAPI1	0.881±0.156	0.816±0.198	0.770±0.097	0.816±0.156	0.341	0.417	0.109	0.971
Right AAPI2	0.993±0.199	1.090±0.291	0.990±0.179	1.028±0.228	0.778	0.740	0.962	0.481
p				<0.001				
Right AARI1	0.576±0.049	0.560±0.112	0.528±0.044	0.552±0.077	0.291	0.475	0.070	0.796
Right AARI2	0.627±0.083	0.676±0.103	0.627±0.066	0.645±0.086	0.241	0.315	0.837	0.089
p				<0.001				
Right AAMV1	85.43±7.72	78.70±16.07	84.30±17.28	82.52±14.68	0.432	0.161	0.962	0.436
Right AAMV2	64.43±17.55	58.10±24.34	44.70±15.38	54.78±20.65	0.247	0.364	0.088	0.529
p				<0.001				
Right AHPI1	1.283±0.454	1.143±0.323	0.933±0.119	1.108±0.334	0.228	0.740	0.114	0.211
Right AHPI2	1.326±0.462	1.425±0.391	1.294±0.267	1.353±0.364	0.620	0.536	0.681	0.400
p				<0.001				
Right AHRI1	0.683±0.086	0.645±0.088	0.602±0.064	0.640±0.083	0.222	0.475	0.091	0.315
Right AHRI2	0.691±0.049	0.791±0.131	0.719±0.106	0.739±0.110	0.190	0.070	0.918	0.211
p				0.002				
Right AHMV1	46.14±4.84	46.00±8.97	44.00±8.09	45.35±7.52	0.793	0.740	0.837	0.549
Right AHMV2	40.86±7.65	37.30±9.55	32.89±7.52	36.73±8.67	0.104	0.417	0.042	0.182
p				<0.001				
Left RPI1	0.964±0.235	0.929±0.204	0.814±0.136	0.896±0.191	0.376	0.432	0.268	0.383
Left RPI2	1.062±0.213	1.084±0.185	0.880±0.167	1.003±0.200	0.088	0.876	0.106	0.053
p				0.001				
Left RRI1	0.610±0.078	0.591±0.078	0.547±0.070	0.580±0.076	0.350	0.755	0.202	0.318
Left RRI2	0.618±0.055	0.657±0.079	0.586±0.087	0.620±0.079	0.117	0.268	0.343	0.128
p				0.012				
Left RMV1	64.00±6.04	65.57±12.01	66.57±7.52	65.53±8.73	0.768	0.755	0.530	0.710
Left RMV2	53.80±6.80	47.14±14.51	47.00±7.37	48.84±10.38	0.432	0.639	0.268	0.456
p				0.001				
Left AAPI1	0.918±0.143	0.894±0.264	0.786±0.091	0.860±0.185	0.289	0.639	0.048	0.902
Left AAPI2	0.960±0.239	1.069±0.309	0.963±0.191	1.001±0.244	0.755	0.639	0.755	0.535
				0.007				
Left AARI1	0.590±0.048	0.583±0.146	0.546±0.053	0.571±0.095	0.370	0.755	0.106	0.535
Left AARI2	0.674±0.206	0.657±0.078	0.616±0.069	0.646±0.117	0.688	0.755	1.000	0.383
p				0.022				
Left AAMV1	80.20±9.15	0.85.29±15.95	83.57±12.29	83.32±12.57	0.806	0.530	0.755	0.902
Left AAMV2	60.60±7.57	62.29±26.09	56.14±6.87	59.58±16.22	0.107	0.876	0.202	0.318
p				<0.001				
Left AHPI1	1.140±0.273	1.164±0.304	0.886±0.111	1.055±0.263	0.086	0.876	0.149	0.038
Left AHPI2	1.294±0.491	1.319±0.281	1.201±0.260	1.269±0.324	0.653	0.639	0.876	0.383
p				0.011				
Left AHRI1	0.648±0.073	0.644±0.080	0.636±0.167	0.642±0.112	0.428	0.755	0.343	0.318
Left AHRI2	0.664±0.103	0.776±0.158	0.691±0.109	0.715±0.130	0.367	0.343	0.755	0.209
p				0.038				
Left AHMV1	43.40±4.98	47.29±8.67	44.29±3.35	45.16±6.10	0.420	0.343	1.000	0.259
Left AHMV2	39.00±7.18	35.71±12.54	39.14±9.62	37.84±9.87	0.967	0.755	1.000	1.000

	MTX / (n=7)	IFX / (n=10)	TCZ / (n=10)	All patients / (n=27)	p MTX vs. IFX vs. TCZ	p MTX vs. IFX	p MTX vs. TCZ	p IFX vs TCZ
p				0.002				
Basilar PI1	1.002±0.176	0.925±0.176	0.874±0.171	0.933±0.178	0.133	0.265	0.046	0.425
Basilar PI2	1.246±0.534	1.001±0.392	0.811±0.149	1.017±0.440	0.003	0.104	0.001	0.037
p				0.986				
Basilar MV1	44.93±17.89	41.71±10.48	44.47±9.24	43.72±12.79	0.541	0.635	0.451	0.331
Basilar MV2	44.14±21.11	31.93±10.51	35.87±5.11	37.28±14.38	0.127	0.077	0.425	0.134
p				<0.001				

Abbreviations: index 1: baseline study; index 2: follow-up study; RPI: resting pulsatility index; RRI: resting resistance index; RMV: resting mean velocity; AAPI: after apnea pulsatility index; AARI: after apnea resistance index; AAMV: after apnea mean velocity; AHPI: after hyperventilation pulsatility index; AHRI: after hyperventilation resistance index; AHMV: after hyperventilation mean velocity, PI: pulsatility index; MV: mean velocity; MTX: methotrexate; IFX:infliximab; TCZ: tocilizumab

Table S7. TCD parameter changes with p-values in all patients.

Previous study	Value	Present study	Value	p-value
Right RPI1	0.908±0.187	Right RPI2	1.062±0.229	<0.001
Right RRI1	0.582±0.069	Right RRI2	0.639±0.086	0.001
Right RMV1	65.74±10.96	Right RMV2	48.56±11.79	<0.001
Right AAPI1	0.816±0.156	Right AAPI2	1.028±0.228	<0.001
Right AARI1	0.552±0.077	Right AARI2	0.645±0.086	<0.001
Right AAMV1	82.52±14.68	Right AAMV2	54.78±20.65	<0.001
Right AHPI1	1.108±0.334	Right AHPI2	1.353±0.364	<0.001
Right AHRI1	0.640±0.083	Right AHRI2	0.739±0.110	0.002
Right AHMV1	45.35±7.52	Right AHMV2	36.73±8.67	<0.001
Left RPI1	0.896±0.191	Left RPI2	1.003±0.200	0.001
Left RRI1	0.580±0.076	Left RRI2	0.620±0.079	0.012
Left RMV1	65.53±8.73	Left RMV2	48.84±10.38	0.001
Left AAPI1	0.860±0.185	Left AAPI2	1.001±0.244	0.007
Left AARI1	0.571±0.095	Left AARI2	0.646±0.117	0.022
Left AAMV1	83.32±12.57	Left AAMV2	59.58±16.22	<0.001
Left AHPI1	1.055±0.263	Left AHPI2	1.269±0.324	0.011
Left AHRI1	0.642±0.112	Left AHRI2	0.715±0.130	0.038
Left AHMV1	45.16±6.10	Left AHMV2	37.84±9.87	0.002
Basilar PI1	0.933±0.178	Basilar PI2	1.017±0.440	0.986
Basilar MV1	43.72±12.79	Basilar MV2	37.28±14.38	<0.001

Abbreviations: RPI: resting pulsatility index; RRI: resting resistance index; RMV: resting mean velocity; AAPI: after apnea pulsatility index; AARI: after apnea resistance index; AAMV: after apnea mean velocity; AHPI: after hyperventilation pulsatility index; AHRI: after hyperventilation resistance index; AHMV: after hyperventilation mean velocity, PI: pulsatility index; MV: mean velocity;

Table S8. Complete cerebrovascular reserve capacity values in RA patients.

	MTX / (n=7)	IFX / (n=10)	TCZ / (n=10)	All patients / (n=27)	p MTX vs. IFX vs. TCZ	p MTX vs. IFX	p MTX vs. TCZ	p IFX vs TCZ
Right MCA CRC (2017)	24.14±11.28	29.40±17.44	28.10±10.41	27.56±13.29	0.640	0.601	0.364	0.684
Right MCA CRC (2023)	15.29±14.95	23.80±22.13	26.70±10.46	22.67±16.74	0.224	0.475	0.070	0.436
p	0.091	0.102	0.465	0.013				
Left MCA CRC (2017)	25.40±9.86	31.00±20.34	26.00±8.00	27.60±13.46	0.984	0.755	1.000	0.867
Left MCA CRC (2023)	12.80±6.94	29.43±25.38	22.50±17.86	22.50±19.33	0.479	0.268	0.435	0.867
p	0.043	0.917	0.612	0.144				

Abbreviations: CRC: cerebrovascular reserve capacity; RA: rheumatoid arthritis; MTX: methotrexate; IFX: infliximab; TCZ: tocilizumab; MCA: middle cerebral artery.

Table S9. Detailed brain MRI findings at baseline and follow-up.

	MTX / (n=14)	IFX / (n=14)	TCZ / (n=15)	All patients / (n=43)	p MTX vs. IFX vs. TCZ	p MTX vs. IFX	p MTX vs. TCZ	p IFX vs TCZ
Undergone Brain MRI1 / Missing Brain MRI1	12 / 2	13 / 1	13 / 2	38 / 5	0.814	1.000	1.000	1.000
Undergone Brain MRI2 Missing Brain MRI2	12 / 2	12 / 2	13 / 2	37 / 6	0.996	1.000	1.000	1.000
Lacunar laesion1 / 0 no / 1 one / 2 few / 3 many	2 / 3 / 5 / 2	9 / 0 / 1 / 3	9 / 0 / 2 / 2	20 / 3 / 8 / 7				
Lacunar laesion2 / 0 no / 1 one / 2 few / 3 many	2 / 2 / 3 / 5	4 / 1 / 3 / 4	9 / 1 / 1 / 2	15 / 4 / 7 / 11				
Lacunar score1 / 0-1 / 2-3	5 / 7	9 / 4	9 / 4	23 / 15	0.165	0.238	0.238	1.000
Lacunar score2 / 0-1 / 2-3	4 / 8	5 / 7	10 / 3	19 / 18	0.028	0.673	0.047	0.111
p				0.500				
non-lacunar emollition1 / 1 – exist / 0 – none	0 / 12	1 / 12	3 / 10	4 / 34	0.157	1.000	0.220	0.593
non-lacunar emollition2 / 1 – exist / 0 - none	1 / 11	2 / 10	3 / 10	6 / 31	0.606	1.000	0.593	1.000
p				0.500				
Atrophy1 / 1 – exist / 0 – none	5 / 7	3 / 10	2 / 11	10 / 28	0.312	0.411	0.202	1.000
Atrophy2 / 1 – exist / 0 – none	6 / 6	4 / 8	3 / 10	13 / 24	0.366	0.680	0.226	0.673
p				0.063				

Abbreviations: 1: baseline study; index 2: follow-up study; MRI: magnetic resonance imaging; MTX – methotrexate; IFX – infliximab; TCZ – tocilizumab. index
